# Mastitis Pathogens with High Virulence in a Mouse Model Produce a Distinct Cytokine Profile *In Vivo*

**DOI:** 10.3389/fimmu.2016.00368

**Published:** 2016-09-22

**Authors:** Carl-Fredrik Johnzon, Karin Artursson, Robert Söderlund, Bengt Guss, Elin Rönnberg, Gunnar Pejler

**Affiliations:** ^1^Department of Anatomy, Physiology and Biochemistry, Swedish University of Agricultural Sciences, Uppsala, Sweden; ^2^National Veterinary Institute (SVA), Uppsala, Sweden; ^3^Department of Biomedical Science and Veterinary Public Health, Swedish University of Agricultural Sciences, Uppsala, Sweden; ^4^Department of Medical Biochemistry and Microbiology, BMC, Uppsala University, Uppsala, Sweden

**Keywords:** mastitis, bovine, inflammation mediators, cytokines, chemokines, bacterial infections

## Abstract

Mastitis is a serious medical condition of dairy cattle. Here, we evaluated whether the degree of virulence of mastitis pathogens in a mouse model can be linked to the inflammatory response that they provoke. Clinical isolates of *Staphylococcus aureus* (*S. aureus*) (strain 556 and 392) and *Escherichia coli* (*E. coli*) (676 and 127), and laboratory control strains [8325-4 (*S. aureus*) and MG1655 (*E. coli*)], were injected i.p. into mice, followed by the assessment of clinical scores and inflammatory parameters. As judged by clinical scoring, *E. coli* 127 exhibited the largest degree of virulence among the strains. All bacterial strains induced neutrophil recruitment. However, whereas *E. coli* 127 induced high peritoneal levels of CXCL1, G-CSF, and CCL2, strikingly lower levels of these were induced by the less virulent bacterial strains. High concentrations of these compounds were also seen in blood samples taken from animals infected with *E. coli* 127, suggesting systemic inflammation. Moreover, the levels of CXCL1 and G-CSF, both in the peritoneal fluid and in plasma, correlated with clinical score. Together, these findings suggest that highly virulent clinical mastitis isolates produce a distinct cytokine profile that shows a close correlation with the severity of the bacterial infection.

## Introduction

Mastitis is the most costly disease in the dairy industry worldwide, incurring economic losses in terms of reduced milk yield, poorer milk quality, and treatment costs (1, 2). Mastitis is defined as an inflammation of the mammary gland, typically the response to an intramammary infection (3, 4). A mastitis case is categorized as either clinical or subclinical. Cows with clinical mastitis present visually recognizable symptoms, such as milk abnormalities and visible changes in the udder. Rapidly developing cases are termed acute clinical mastitis. Subclinical mastitis is marked by the absence of macroscopically visual symptoms, though milk abnormalities or udder changes may occur transiently in a minority of cases. Subclinical cases that persist for at least 2 months are termed chronic. Intramammary infections are typically of bacterial origin and the responsible pathogens are broadly categorized as either contagious (spread between udder quarters or cows) or environmental (opportunistic pathogens) (5, 6). Staphylococci, streptococci, and coliform bacteria constitute the majority of etiological mastitis agents (7, 8).

The immune response toward an intramammary infection in cows is greatly influenced by the species of the invading bacterium. *Escherichia coli* (*E. coli)* commonly causes rapid and powerful immune responses (i.e., acute clinical mastitis), whereas *Staphylococcus aureus* (*S. aureus*) infections are associated with chronic responses (3). The course of the disease is also influenced by the strain and bacterial virulence factor, as well as by host and environmental factors. In the case of *E. coli*, it has been shown that strains possessing or having upregulated expression of factors that enable adhesion, invasion, motility (*CheA, Tsr*, and *Tar*), and iron acquisition (*iroN* and *sitA*) are more likely to cause persistent rather than transient intramammary infections (9–11). Cows infected with more common *S. aureus* genotypes experienced faster resolution of the inflammatory response than cows infected with less common genotypes (12), possibly because the less common genotypes possessed virulence factors enabling persistent infections. Other studies have found that certain genes are more common in isolates derived from persistent infection. For example, pyrogenic toxin superantigens (PTSAg), enterotoxin-encoding genes (*sed* and *sej*), and a penicillin resistance gene (*blaZ*) were more common in persistent strains (13, 14).

The objective of the current study was to differentiate the virulence of a set of acute clinical mastitis isolates using a mouse infection model. Non-mammary mouse models have previously been used to test the immune response toward mastitis-causing bacterial strains, in particular with the aim of defining novel vaccination strategies for mastitis (15–18). However, such models have not been extensively used to evaluate the inflammatory response toward mastitis pathogens. The current study used an acute intraperitoneal infection model. *S. aureus* and *E. coli* were selected by virtue of being common mastitis pathogens (8) and for representing the two mastitis pathogen classes – contagious (*S. aureus*) and environmental (*E. coli*). We demonstrate strain-dependent variations in virulence in the mouse model, with highly virulent *E. coli* inducing a defined set of inflammatory compounds including G-CSF and CXCL1. Moreover, the levels of these compounds correlated significantly with clinical outcome, suggesting the possibility to further evaluate G-CSF and CXCL1 as therapeutic targets, therapeutics, or biomarkers in mastitis.

## Materials and Methods

### Mice

Female B6JBOM (C57BL/6) mice (11–17 weeks old) were purchased from Taconic. After arrival, the mice were allowed a 1-month period of acclimatization prior to use. The mice were moved to an infection laboratory unit 4 days prior to the start of an experiment. During the entire period, the mice were fed rodent chow and water *ad libitum*. The animals had access to enrichment in the form of paper and paper houses. All animal experiments were approved by the local ethical committee (Uppsala Djurförsöksetiska Nämnd; no C85/14) and were performed in accordance with relevant guidelines and regulations.

### Bacterial Strains

The clinical mastitis isolates 556 (*S. aureus*), 392 (*S. aureus*), 676 (*E. coli*), and 127 (*E. coli*) were obtained from the collection at the Swedish National Veterinary Institute (SVA). These strains were originally isolated from Swedish dairy cows during a national survey (8). Samples were only taken from lactating cows with acute clinical mastitis that had not undergone treatment with antimicrobials during the previous 30 days, had macroscopic changes in the milk in at least one udder quarter, had no previous record of clinical mastitis during the current lactation period, and a somatic cell count at the latest monthly milking of <200,000 cells/ml.

As controls for the clinical isolates, we used laboratory strains of *S. aureus* (8325-4) and *E. coli* (MG1655). The *S. aureus* strain 8325-4 (19) was obtained from the collection at the Department of Microbiology at the Swedish University of Agricultural Sciences. The *E. coli* strain MG1655 was a kind gift from Diarmaid Hughes (Department of Medical Biochemistry and Microbiology, Uppsala University).

### Genotyping

DNA was extracted from *E. coli* and *S. aureus* colony material collected from horse blood agar plates, using the DNeasy Blood & Tissue Kit (Qiagen, Hilden, Germany), according to the manufacturer’s instructions. Genotyping was performed using DNA microarrays. The Identibac *S. aureus* Genotyping System (Alere, Jena, Germany) was used to genotype the *S. aureus* isolates. The system detects 333 *S. aureus* genes and gene variants relevant for typing as well as resistance and virulence attributes. *E. coli* isolates were characterized using the Identibac Ec System (Alere), detecting 124 *E. coli* virulence genes and gene variants as well as control genes. The assays were performed according to the manufacturer’s instructions. Hybridization results were detected by an ArrayMate Reader (Alere), and signals were registered as positive, ambiguous, or negative for each gene or gene variant.

### Bacterial Cultures

Bacterial strains were streaked onto horse blood agar (*S. aureus*) (SVA, Uppsala, Sweden) or nutrient agar plates (*E. coli*) (Oxoid, Basingstoke, UK). Irrespective of species, the plates were incubated at 37°C for 24 h, after which colonies were picked and used to inoculate 20 ml of Tryptic Soy Broth (TSB; BD, Franklin Lakes, NJ, USA). Following incubation at 37°C for 16–17 h, 200 μl of overnight culture was transferred to 20 ml fresh TSB and incubated at 37°C until the OD_600_ reached 0.5. In preparation for injection, bacteria were washed twice in PBS (SVA; taken to room temperature) and finally resuspended in a volume of fresh TSB yielding a 1:1 dilution.

### Generation Time

The generation time for each strain was determined from the increase in OD_600_ during the exponential phase. Briefly, the strains were incubated in TSB at 37°C, and the OD_600_ was measured every 30th minute until entry into the stationary phase. The assay was performed in triplicates.

### *In vivo* Infection & Clinical Scoring

Mice were injected intraperitoneally with 100 μl TSB medium containing bacteria (~2 × 10^8^ CFU of *E. coli* or ~5 × 10^8^ CFU of *S. aureus*). Body weight was monitored prior to injection. After 24 h, the general condition of the mice was assessed by clinical scoring (Table 1), followed by weighing. The mice were subsequently euthanized, and samples were taken in the following order: blood *via* the eye, peritoneal lavage with 5 ml cold PBS, mesenteric lymph nodes, and spleens. The lymph nodes and spleens were weighed. Presence of *E. coli* in the blood was determined by streaking a 10 μl aliquot of blood onto MacConkey agar (Oxoid). The colony-forming unit count (CFU) in the peritoneal lavage fluid was determined by plating a 100 μl aliquot onto horse blood agar (*S. aureus*) or nutrient agar/MacConkey agar (*E. coli*). The cells in peritoneal lavage fluid were counted. Cytospins were prepared from an aliquot of the peritoneal cells and were stained with May-Grünwald/Giemsa, followed by differential counting. Finally, the remaining cells in the peritoneal lavage fluid were pelleted by centrifugation; the supernatant was collected and stored at −20°C.

**Table 1:** **Clinical scoring**.

Clinical Score^a^	Criteria
1	Active, responsive, and no signs of any illness
2	Slower in reaction to stimuli, but otherwise active and healthy
3	Slow and lethargic, but still active
4	Inactive but still responsive to stimuli, albeit slowly
5	Inactive and non-responsive to any stimuli
Additional scores of 0.5 were added cumulatively based on the presence of:	Ocular changes [dry encrusted eye(s), pus covered eyes or a grayish opaque film] Intestinal disturbances (loose fecal pellets) Shivering

*^a^Note that the maximal score is 6.5 (main clinical scores 1–5 + additional scores of 0.5)*.

### Cytokine Arrays & ELISAs

Cytokine levels in the peritoneal lavage fluid were determined using RayBio^®^ Mouse Inflammation Array C1 (RayBiotech, Inc., Norcross, GA, USA), according to the manufacturer’s instructions. Equal volumes of peritoneal lavage fluid from five mice were pooled to give the volume required for one membrane. The integrated density of the upregulated cytokines was quantified using ImageJ software and was normalized using the positive controls on each filter. The density of each spot was then compared with negative controls (peritoneal lavage fluid from non-infected animals); results are given as % density in comparison with the negative control. ELISAs were performed on peritoneal lavage fluid and blood plasma for CCL2 (Ready-SET-Go; eBioscience), CXCL1, and G-CSF (PeproTech), according to the manufacturers’ instructions.

### Statistical Analysis

Data are shown as means ± SD. Statistical analyses were performed by using GraphPad Prism 6.0 (GraphPad Software), using one-way ANOVA without matching and Fisher’s LSD *post hoc* test. Correlation analysis was performed using non-parametric Spearman correlation with a two-tailed P-value.

## Results

### Genotype and Growth Characteristics of Mastitis Pathogens

The clinical isolates used in this study were all derived from the milk of cows suffering from acute clinical mastitis. The clinical isolates were genotyped (see Tables S1 and S2 in Supplementary Material). As shown in Table S1 in Supplementary Material, the *S. aureus* strains 556 and 392 clinical isolates shared a high degree of similarity in terms of virulence factors. However, they differed in terms of enterotoxins, with strain 392 harboring genes encoding enterotoxins, whereas *S. aureus* 556 did not. The *E. coli* clinical isolate 676 carried genes encoding cytotoxic necrotizing factor (*cnf*), cytolethal distending toxin (*cdt*), as well as the F17 fimbriae. This trio of virulence factors encoded on the same plasmid (pVir) is characteristic of type 2 necrotoxic *E. coli* (NTEC-2), which has been reported to frequently colonize the gastrointestinal tract of healthy ruminants [reviewed by De Rycke et al. (20)], but also been shown to cause both diarrhea and invasion of the blood stream in newborn calves (21), dependent only on factors encoded on the Vir plasmid (22). The *E. coli* 127 isolate lacked all virulence factors represented in the microarray system (Table S2 in Supplementary Material).

**Figure 1:**
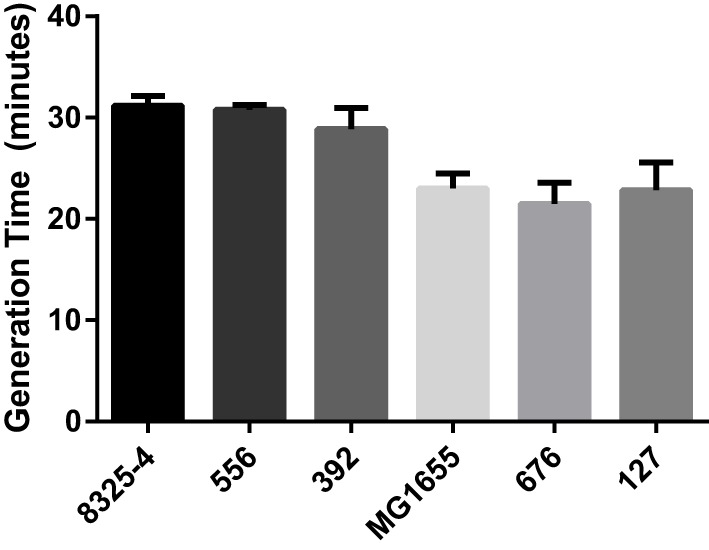
**Generation times of bacterial strains**. Generation times for the *Staphylococcus aureus* strains (8325-4, 556, 392) and the *Escherichia coli* strains (MG1655, 676, 127) were determined from the exponential phase measured in TSB and at OD_600_.

The bacterial growth rate has previously been suggested to influence mastitis strain virulence (3). In order to assess whether differences in growth rate of these bacterial strains could influence their virulence, the generation time for each strain was determined in TSB at 37°C. As shown in Figure 1, all strains of each species had virtually identical generation times, approximately 30 min for *S. aureus* and 22 min for *E. coli*.

### Mice Infected with *E. coli* 127 Exhibit Severe Clinical Symptoms

**Figure 2:**
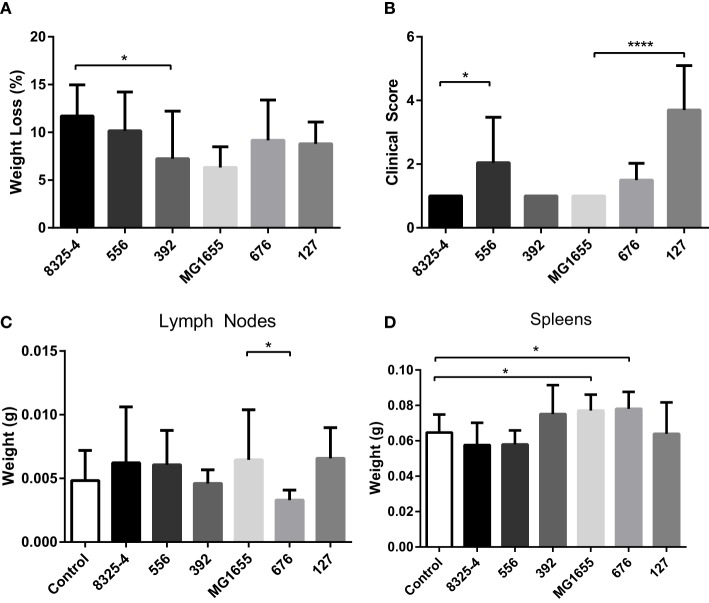
**Effect of intraperitoneal bacterial infection on weight loss and clinical outcome**. Mice were injected intraperitoneally with *Staphylococcus aureus* (8325-4, 556, 392) or *Escherichia coli* (MG1655, 676, 127). **(A)** After 24 h, weight loss of mice was monitored. **(B)** Severity of infection was monitored using clinical scoring as described in Table 1. **(C,D)** The weights of mesenteric lymph nodes **(C)** and spleens **(D)** were assessed. Data represent mean ± SD. *p < 0.05; ****p < 0.0001 (*n* = 4–10).

To investigate differences in clinical outcome for the selected strains, a mouse intraperitoneal infection model was used. Clinical outcome was assessed by measuring weight loss and by scoring clinical signs 24 h after infection. The mesenteric lymph nodes and spleens were also collected and weighed. As shown in Figure 2A, the weight loss of the mice in response to the different bacterial strains was relatively similar, with averages ranging from 6–12% over 24 h. In addition to monitoring clinical outcome by weight changes, we also implemented a clinical score system ranging from 1 to 5, where 1 represented animals with normal activity and 5 represented inactive, unresponsive animals (Table 1). Additional scores of 0.5 were added cumulatively for the presence of other clinical signs (ocular changes, intestinal disturbances, and shivering; see Table 1). As displayed in Figure 2B, the *E. coli* strain 127 caused the highest increase in clinical score out of all the bacterial strains tested. Moreover, mice infected with *S. aureus* 556 developed higher clinical scores than animals infected with the control *S. aureus* strain (8325-4).

None of the assessed bacterial strains caused a significant increase in the weight of the mesenteric lymph nodes in comparison with non-infected controls (Figure 2C). However, we noted that lymph nodes taken from mice infected with the *E. coli* strain 676 weighed less than those from mice infected with *E. coli* MG1655 (Figure 2C). Infection with either of the *S. aureus* strains did not cause any significant effects on spleen weight (Figure 2D). In contrast, infection with *E. coli* MG1655 or *E. coli* 676 caused small, yet significant, increase in the weight of the spleens. Notably though, infection with the *E. coli* strain 127 did not result in increased spleen weight (Figure 2D).

**Figure 3:**
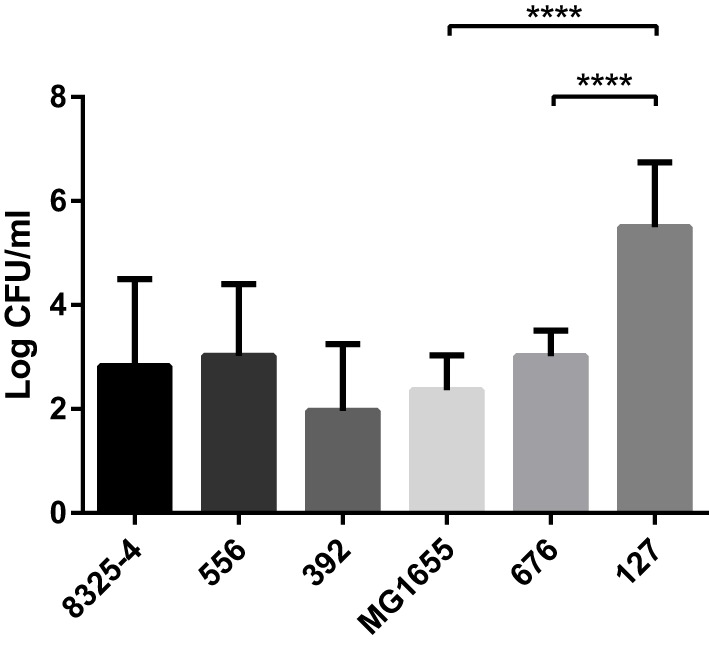
**Presence of bacteria in the peritoneal cavities of infected mice**. Mice were injected intraperitoneally with *Staphylococcus aureus* (8325-4, 556, 392) or *Escherichia coli* (MG1655, 676, 127). After 24 h, the presence of persistent bacteria (colony-forming units; CFU) in the peritoneal lavage fluid was determined. Data represent mean ± SD. ****p < 0.0001 (*n* = 4–10).

To monitor any differences in bacterial persistence *in vivo*, peritoneal lavage was performed on the mice after euthanasia, followed by plating the fluid onto agar plates and quantification of the CFU. Mice infected with the clinical *E. coli* isolate 127 exhibited a poor bacterial clearance compared with the laboratory strain MG1655 (Figure 3). In particular, the strain 127 persisted at very high numbers after 24 h in the mice. In contrast, the clinical *S. aureus* isolates 556 and 392 did not yield higher CFU numbers than did the laboratory strain 8325-4.

Cows afflicted with acute coliform mastitis have previously been shown to frequently develop bacteremia originating from the mammary infection (23, 24). To determine whether any of the *E. coli* strains assessed here could spread outside the peritoneal cavity, blood from infected mice was streaked onto MacConkey agar and incubated at 37°C for 24 h. As displayed in Table 2, *E. coli* strain 127 was present in these blood streaks, while MG1655 and 676 were not.

**Table 2:** **Bacteremia after *Escherichia coli* infection**.

Strain	Bacteria detected in blood
MG1655	0/5
676	0/5
127	5/5

*Isolate 127 but not 676 or the laboratory strain MG1655 causes bacteremia. Mice were injected intraperitoneally with *Escherichia coli* (MG1655, 676, 127). After 24 h mice were euthanized. Blood samples were assessed for bacteremia*.

### Mastitis Pathogens Cause Leukocyte Recruitment into the Peritoneal Cavity

**Figure 4:**
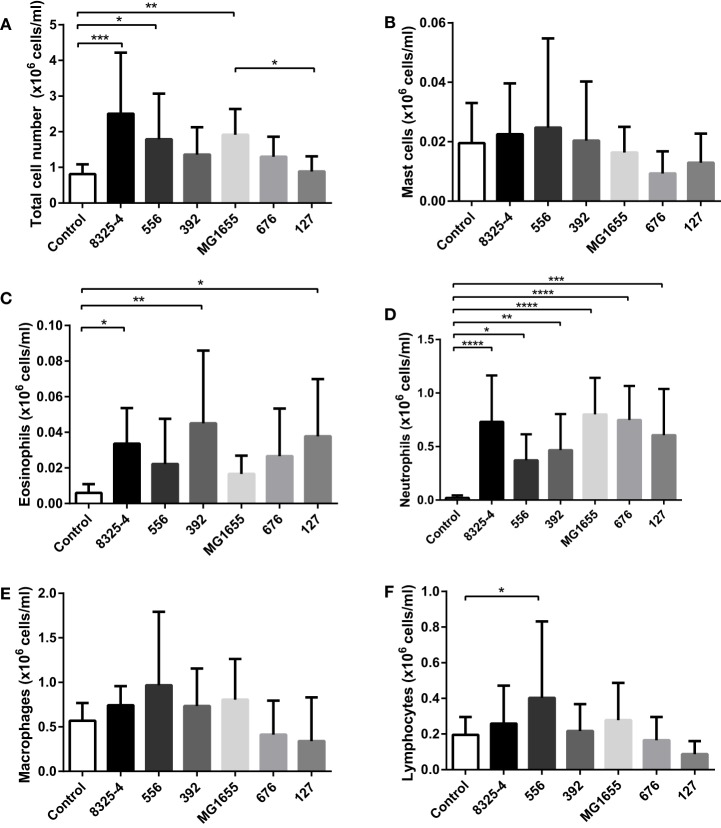
**Effect of bacterial infection on peritoneal leukocyte populations**. Mice were injected intraperitoneally with *Staphylococcus aureus* (8325-4, 556, 392) or *Escherichia coli* (MG1655, 676, 127). After 24 h, peritoneal lavage was performed and the **(A)** number of total cells was quantified; differential counting of mast cells **(B)**, eosinophils **(C)**, neutrophils **(D)**, macrophages **(E)**, and lymphocytes **(F)** was performed after May-Grünwald/Giemsa staining of cytospin slides. Data represent mean ± SD. *p < 0.05; **p < 0.01; ***p < 0.001; ****p < 0.0001 (*n* = 6–10).

Next, we investigated whether the difference in clinical signs in response to the various bacterial strains tested could be associated with differences in inflammatory parameters. To this end, peritoneal cell populations from the infected mice were collected, followed by the assessment of total cell counts and differential cell counting after May-Grünwald/Giemsa staining of cytospin slides. As seen in Figure 4A, infection with *S. aureus* 8325-4, *S. aureus* 556, and *E. coli* MG1655 caused a significant increase of the total cell counts in the peritoneal cavity. In contrast, there was only a trend of increased total cell counts after infection with *S. aureus* 392 and *E. coli* 676, and no effect on total cell counts of the peritoneal cavity were seen after infection with *E. coli* 127. In response to all of the tested bacterial strains, a significant influx of neutrophils into the peritoneal cavity was seen (Figure 4D). In contrast, the numbers of peritoneal mast cells (Figure 4B) or macrophages (Figure 4E) were not affected by any of the bacteria, and there was no increase in the lymphocyte population in response to any of the bacterial strains, except for a modest increase in lymphocyte numbers in response to *S. aureus* 556 (Figure 4F). Eosinophil populations were only marginally affected by infection with the bacterial strains used (Figure 4C), although it was noted that infection with *S. aureus* 392 and *E. coli* 127 caused a slight, yet significant, increase in peritoneal eosinophil numbers (Figure 4C).

### *E. coli* Strain 127 Causes a Local and a Systemic Cytokine Response

**Figure 5:**
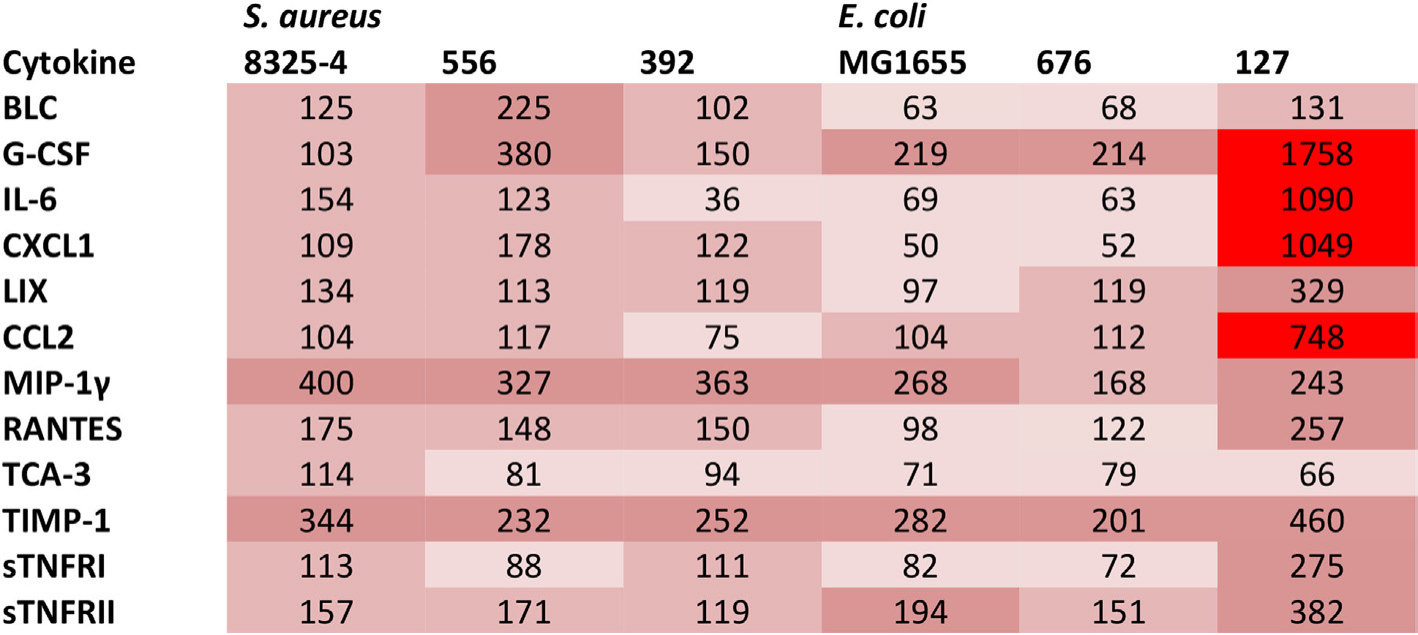
**Presence of cytokines in the peritoneal fluids after bacterial infection**. Mice were injected intraperitoneally with *Staphylococcus aureus* (8325-4, 556, 392) or *Escherichia coli* (MG1655, 676, 127). After 24 h, peritoneal lavage was performed. The presence of pro-inflammatory cytokines in peritoneal lavage fluid was determined by using an antibody-based array. The figure represents quantification of the cytokine array data by densitometry using ImageJ. Data are given as % integrated density in comparison to negative controls (plasma from non-infected animals). Levels of expression correlate with the intensity of red color.

**Figure 6:**
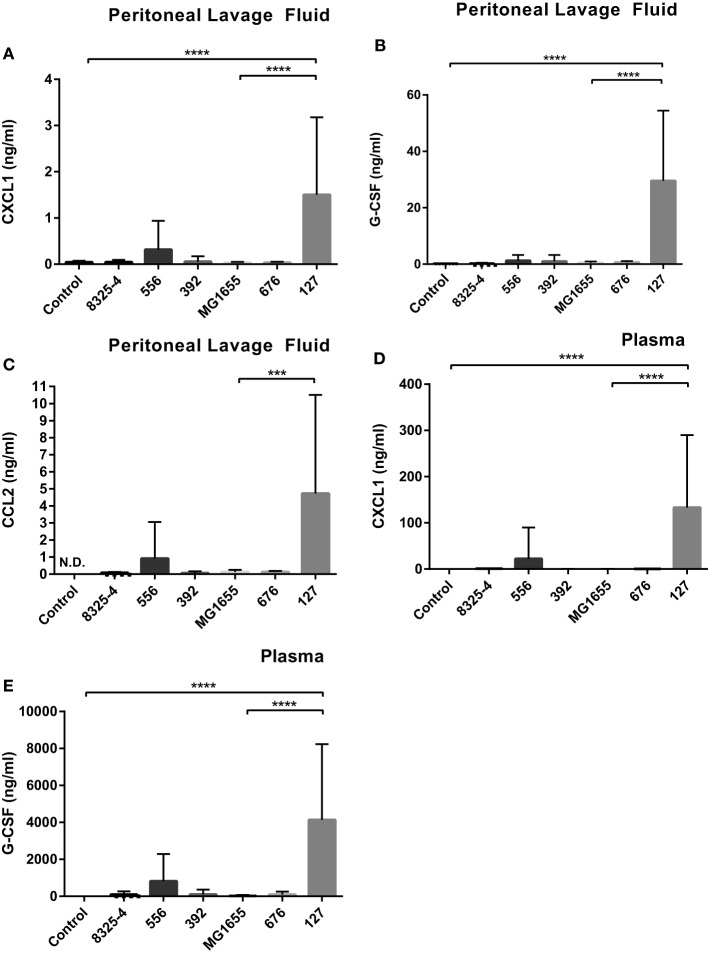
***E. coli* 127 induces high levels of G-CSF, CXCL1, and CCL2**. Mice were injected intraperitoneally with *Staphylococcus aureus* (8325-4, 556, 392) or *Escherichia coli* (MG1655, 676, 127). After 24 h, peritoneal lavage fluid and blood plasma samples were collected. **(A–C)** The presence of CXCL1 **(A)**, G-CSF **(B)**, and CCL2 **(C)** in peritoneal lavage fluid was determined by using ELISA. **(D,E)** The presence of CXCL1 **(D)** and G-CSF **(E)** in plasma was determined by using ELISA. Data represent mean ± SD. ***p < 0.001: *p* < 0.0001 (*n* = 6–10).

**Figure 7:**
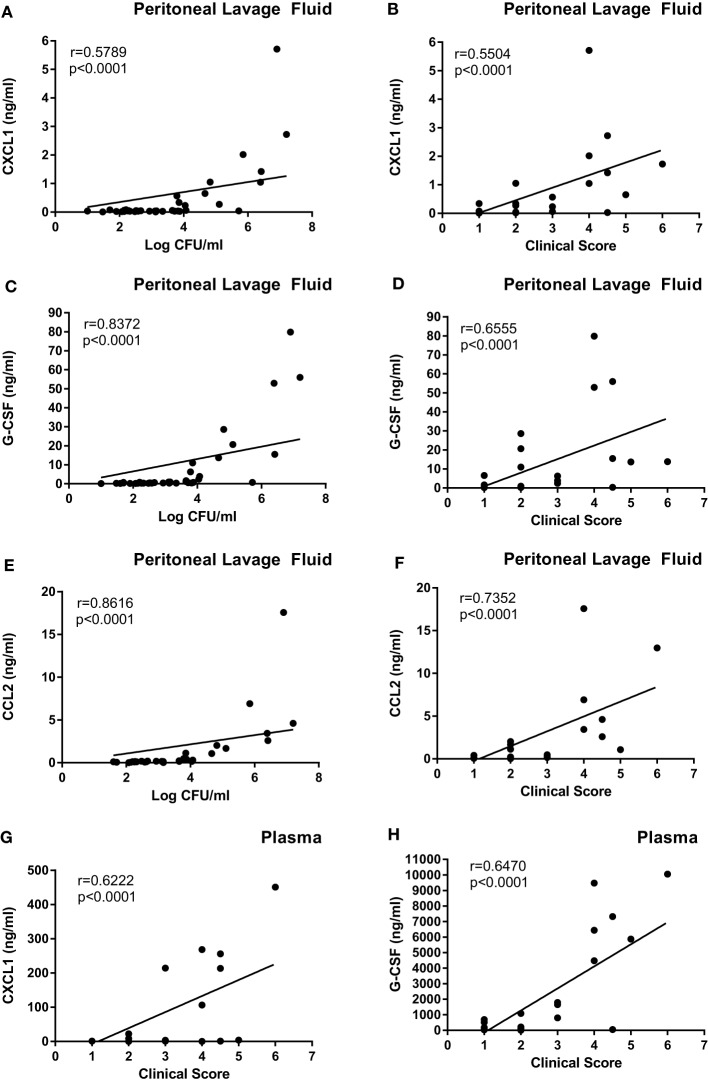
**The levels of G-CSF, CXCL1, and CCL2 correlate with clinical outcome and with bacterial burden**. Mice were injected intraperitoneally with *Staphylococcus aureus* (8325-4, 556, 392) or *Escherichia coli* (MG1655, 676, 127). **(A,C,E)** Correlation between bacterial counts (CFU) and CXCL1 **(A)**, G-CSF **(C)**, and CCL2 **(E)** levels in the peritoneal lavage fluid. **(B,D,F)** Correlation between clinical score and levels of CXCL1 **(B)**, G-CSF **(D)**, and CCL2 **(F)** in the peritoneal lavage fluid. **(G,H)** Correlation between clinical score and levels of CXCL1 **(G)** and G-CSF **(H)** in plasma.

In order to further investigate if the difference in clinical outcome of the different bacterial strains could be related to inflammatory parameters, we assessed differences in the cytokine output in response to the respective bacteria. We used an inflammatory cytokine array as a screening method for this purpose. As shown in Figure S1 in Supplementary Material (shown for the *E. coli* strains) and Figure 5 (quantification by densitometry of data from all bacteria), several inflammatory cytokines/compounds were induced to varying extents by the various bacterial strains. However, the most striking induction of cytokines was seen after infection with *E. coli* 127. In particular, there was a dramatic increase in the levels G-CSF, IL-6, CXCL1, and CCL2 in response to this bacterial strain, whereas much lower levels of these compounds were seen in the peritoneal cavity of mice infected with any of the other bacterial strains. To confirm these findings by independent methodology, we used ELISA. As shown in Figures 6A–C, ELISA measurements confirmed a robust induction of CXCL1, G-CSF, and CCL2 in the peritoneal cavity of mice infected with *E. coli* 127 in comparison with infection with any of the other bacteria. However, a trend of upregulation of these compounds was also seen in response to *S. aureus* 556. Notably, there was a strong positive correlation between the bacterial counts in the peritoneal lavage fluid (CFU) and the levels of levels of CXCL1, G-CSF, and CCL2 (Figures 7A,C,E). As mice infected with *E. coli* 127 displayed the most severe clinical signs of infection, we additionally asked whether the levels of these inflammatory compounds showed a correlation with clinical score. Indeed, there was a significant positive correlation between the clinical score and the levels of CXCL1, G-CSF, and CCL2, respectively (Figures 7B,D,F).

Next, we assessed if the induction of inflammatory cytokines in the peritoneal cavity also was reflected by corresponding increases in blood plasma samples, i.e., reflecting a systemic inflammatory response. Indeed, infection with *E. coli* 127 caused a dramatic increase in the levels of G-CSF and CXCL1 in plasma, whereas none of the other bacterial strains tested caused significant upregulation of these compounds (Figures 6D,E). However, a tendency toward upregulated G-CSF and CXCL1 plasma levels was seen after infection with *S. aureus* 556 (Figures 6D,E). To further evaluate the association between the levels of G-CSF and CXCL1 with clinical outcome, we assessed whether levels of plasma G-CSF and CXCL1 showed a correlation with clinical score. As seen in Figures 7G,H, the plasma levels of both of these compounds were positively correlated with the clinical score. Together, these findings suggest that the high virulence of *E. coli* 127 is associated with a dramatic induction of a specific set of inflammatory compounds, such as CXCL1 and G-CSF, and that the levels of these compounds are correlated with clinical outcome and bacterial burden.

## Discussion

The outcome of bovine mastitis is determined by host (25, 26) and pathogen factors (27, 28). It is known that the severity of mastitis varies depending on the species of the causative bacteria [reviewed in Ref. (3)]. Additionally, particular genes encoding virulence factors have been frequently isolated from bacterial strains able to cause more severe or persistent intramammary infections. Although there is some knowledge regarding the virulence factors that are expressed by common mastitis pathogens (see Introduction), there is still only limited knowledge of how mastitis pathogens affect the host immune response, a limiting factor for such research efforts being the challenging task of performing relevant studies in the bovine species. As an alternative, it may be possible to address this issue by performing studies in rodent models, and, here, we aimed to elucidate how mastitis pathogens affect the immune response by using a mouse acute intraperitoneal infection model. Although it can be questioned whether such mouse non-mammary models give an adequate picture of the inflammatory events occurring in mastitis, it should be emphasized that non-mammary models have previously been used for this purpose and have been suggested to represent useful tools for studying mastitis (17, 18, 29, 30).

Among the mastitis pathogens evaluated here, we found that the *E. coli* strain 127 exhibited the highest virulence as judged by clinical scoring. The higher virulence of this strain as opposed to the less virulent *E. coli* strains tested was most likely not due to a higher growth rate, as shown by analysis of the generation times for the different bacterial strains. This finding is, thus, in some contrast to earlier studies, where it has been suggested that the *in vitro* growth rate is predictive of *in vivo* virulence (17, 31). Moreover, *E. coli* 127 did not induce a more profound effect on lymphoid organ weight as compared with the other strains tested. It was also apparent that all of the bacterial strains tested produced a relatively similar inflammatory response in terms of neutrophil recruitment, whereas no significant recruitment of monocytes/macrophages or other leukocytes populations was observed, indicating that the high virulence of *E. coli* 127 is not due to a failure of the host to recruit neutrophils to clear the infection. However, *E. coli* 127 was shown to persist in the peritoneal cavity at markedly higher numbers as compared with the other bacterial strains tested. This suggests that *E. coli* 127 has a higher capability of evading eradication by the host response than have the other tested strains. Alternatively, although not possessing an intrinsically higher growth rate compared with the other *E. coli* strains, *E. coli* 127 may be better adapted than the other strains to maintain growth in the peritoneal milieu. It was also observed that infection with *E. coli* 127 was associated with bacteremia, whereas the other *E. coli* strains tested did not penetrate into the blood. This indicates that infection with *E. coli* 127 results in a systemic infection, well in line with the severe outcome as judged by clinical scoring. In line with these findings, bacteremia is common among cows afflicted with acute coliform mastitis (23, 24, 32). Interestingly, *E. coli* 127 possessed none of the virulence genes represented in the microarray system, suggesting the involvement of other less obvious virulence or survival factors. In contrast, *E. coli* 676 belongs to the NTEC-2 pathotype known to be capable of causing bacteremia in cattle (21, 22), but was less virulent as judged by clinical scoring in the present study.

In order to further investigate the reason(s) behind the differential virulence of the bacterial strains tested, we assessed the cytokine output induced by the various bacterial strains. We, thereby, found that the highly virulent *E. coli* 127 induced a dramatic increase of G-CSF, CXCL1, CCL2, and IL-6, whereas the levels of these were low in peritoneal cavities of mice infected with any of the other bacterial strains. Importantly, we found high levels of such compounds also in the blood plasma, in line with a systemic inflammatory response induced by *E. coli* 127. G-CSF is a growth factor that promotes the survival, proliferation, and differentiation of neutrophils, as well as enhancing the activity of mature neutrophils (33), and we may, thus, envisage that the high induction of G-CSF reflects an attempt by the host to cope with the bacterial infection by promoting the expansion of the neutrophil population. CXCL1 is a chemokine with neutrophil attracting properties (34), and the induction of this chemokine may, thus, also reflect the need for neutrophil recruitment in order to cope with the severe infection caused by *E. coli* 127 (34). In agreement with a role of this chemokine in mastitis, CXCL1 is detectable at the protein level in the bovine mammary gland in response to intramammary lipoteichoic acid (LTA) infusion (35). CCL2 is a chemokine that is chemoattractant for macrophages/monocytes (36). The induction of this chemokine may, thus, suggest a role of macrophages/monocytes in the host response. Of note, bovine mammary epithelial cells and mammary tissue express CCL2 mRNA in response to LPS stimuli (37, 38), in agreement with a link between this chemokine and mastitis. IL-6 is a powerful, pro-inflammatory cytokine with wide impact on the host response against various pathogens (39), and it has been shown previously that IL-6 is elevated during mastitis (40). High levels of this cytokine may, thus, be expected to accompany a severe bacterial infection by mastitis pathogens, such as the one caused by *E. coli* 127.

Altogether, these findings suggest that severe bacterial infection caused by mastitis pathogens results in a distinct profile of induced inflammatory compounds. Most likely, the induction of these compounds may represent a response that is protective for the host, by promoting the recruitment of neutrophils to the site of infection. We may, thus, envisage that treatment of infected subjects with the corresponding compounds may represent a potential therapeutic approach to limit infection. In line with this, attempts to enhance the immune competence of periparturient dairy cows by administrating recombinant G-CSF have been shown to increase the numbers and functional properties of neutrophils (41, 42).

The basis of the high virulence of *E. coli* 127 is intriguing. As judged by our genotyping for the presence of known virulence factors, *E. coli* 127 did not harbor genes coding for additional virulence factors in comparison with *E. coli* 676. The high virulence of *E. coli* 127 is, therefore, most likely due to non-characterized virulence factors/mechanisms. However, the nature of such factors/mechanisms is at present unknown. Interestingly, these findings are in analogy with the results of an extended survey of *E. coli* milk isolates from bovine mastitis, where it was observed that many of the pathogens causing acute clinical mastitis lacked well-characterized virulence factor genes (K. Artursson et al., unpublished results). This notion is also supported by a recent study from Kempf et al. who demonstrated by using a genomic comparative approach that mastitis *E. coli* isolates lacked well-characterized virulence factors (43).

As an alternative to exploiting these findings for therapeutic purposes, we foresee the possibility of using our findings for diagnostic purposes. Clearly, there is a demand for improved tools for diagnosing different grades of mastitis, in particular for detection of subclinical mastitis (44). Based on the findings obtained in this investigation, high levels of G-CSF and CXCL1 may accompany severe, systemic bacterial infection, and it is, thus, possible that levels of G-CSF and/or CXCL1 may be useful diagnostic criteria in mastitis. In agreement with such a notion, we found that the levels of these compounds correlated significantly with severity of infection as judged by clinical scoring. However, further studies are needed to clarify whether induction of these particular pro-inflammatory factors also accompanies bovine mastitis.

## Author Contributions

C-FJ planned and performed experiments, interpreted data, and wrote the manuscript; KA collected bacterial strains, performed genotyping, and contributed to the writing of the manuscript; RS performed genotyping and contributed to the writing of the manuscript; BG contributed to experiments and to the writing of the manuscript; ER planned and performed experiments, interpreted data, and contributed to the writing of the manuscript; GP planned the study, interpreted data, and wrote the manuscript. All authors reviewed the manuscript.

## Conflict of Interest Statement

The authors declare that the research was conducted in the absence of any commercial or financial relationships that could be construed as a potential conflict of interest.
