# Supplementary material for: Mastitis Pathogens with High Virulence in a Mouse Model Produce a Distinct Cytokine Profile In Vivo
Source: Front Immunol. 2016 Sep 22;7:368. doi: 10.3389/fimmu.2016.00368 (PMC5031784; doi:10.3389/fimmu.2016.00368)
Supplement: Supplementary file 1 [file Table_1.PDF]

Supplemental Table 1. Genotyping of *S. aureus* clinical isolates; strain 392 and 556

	S. aureus strain	
**Gene name**	**392**	**556**
Ribos. STAU	Positive	Positive
gapA	Positive	Positive
katA	Positive	Positive
CoA	Positive	Positive
nuc1	Positive	Positive
spa	Positive	Positive
sbi	Positive	Positive
sarA	Positive	Positive
saeS	Positive	Positive
vraS	Positive	Positive
agrI (total)	Positive	Positive
agrB-I	Positive	Positive
agrC-I	Ambiguous	Positive
agrD-I	Positive	Positive
agrII (total)	Negative	Negative
agrB-II	Negative	Negative
agrC-II	Negative	Negative
agrD-II	Negative	Negative
agrIII (total)	Negative	Negative
agrB-III	Negative	Negative
agrC-III	Negative	Negative
agrD-III	Negative	Negative
agrIV (total)	Negative	Ambiguous
agrB-IV	Ambiguous	Positive
agrC-IV	Negative	Negative
hld	Positive	Positive
mecA	Negative	Negative
delta_mecR	Negative	Negative
ugpQ	Negative	Negative
ccrA-1	Negative	Negative
ccrB-1	Negative	Negative
plsSCC (COL)	Negative	Negative
Q9XB68-dcs	Negative	Negative
ccrA-2	Negative	Negative
ccrB-2	Negative	Negative
kdpA-SCC	Negative	Negative
kdpB-SCC	Negative	Negative
kdpC-SCC	Negative	Negative
kdpD-SCC	Negative	Negative
kdpE-SCC	Negative	Negative
mecI	Negative	Negative
mecR	Negative	Ambiguous
xylR	Negative	Ambiguous
ccrA-3	Negative	Negative
ccrB-3	Negative	Negative
merA	Negative	Negative
merB	Negative	Negative
ccrAA (MRSAZH47)_probe 1	Negative	Negative
ccrAA (MRSAZH47)_probe 2	Negative	Negative
ccrC (85-2082)	Negative	Negative
ccrA-4	Negative	Negative
ccrB-4	Negative	Negative
blaZ	Negative	Negative
blaI	Negative	Negative
blaR	Negative	Negative

ermA	Negative	Negative
ermB	Negative	Negative
ermC	Negative	Negative
linA	Negative	Negative
msrA	Negative	Negative
mefA	Negative	Negative
mpbBM	Negative	Negative
vatA	Negative	Negative
vatB	Negative	Negative
vga	Negative	Negative
vgaA	Negative	Negative
vgb	Negative	Negative
aacA-aphD	Negative	Negative
aadD	Negative	Negative
aphA3	Negative	Negative
sat	Negative	Negative
dfrA	Negative	Negative
far1	Negative	Negative
Q6GD50 (fusC)	Negative	Negative
mupR	Negative	Negative
tetK	Negative	Negative
tetM	Negative	Negative
cat	Negative	Negative
cat (pC221)	Negative	Negative
cat (pc223)	Negative	Negative
cat (pMC524)	Negative	Negative
cat (pSBK203R)	Negative	Negative
cfr	Negative	Negative
fexA	Negative	Negative
fosB	Positive	Positive
fosB-plasmid	Negative	Positive
qacA	Negative	Negative
qacC	Negative	Negative
qacC (cons)	Negative	Negative
qacC (equine)	Negative	Negative
qacC (SA5)	Negative	Negative
qacC (Ssap)	Negative	Negative
qacC (ST94)	Negative	Negative
tetEfflux/sdrM	Positive	Positive
vanA	Negative	Negative
vanB	Negative	Negative
vanZ	Negative	Negative
tst1 (consensus)	Negative	Negative
tst1 ("human" allele)	Negative	Negative
tst1 ("bovine" allele)	Negative	Negative
entA	Negative	Negative
entA (320E)	Negative	Negative
entA (N315) / entP	Negative	Negative
entB	Negative	Negative
entC	Negative	Negative
entD	Negative	Ambiguous
entE	Negative	Negative
entG	Positive	Ambiguous
entH	Negative	Negative
entI	Positive	Negative
entJ	Negative	Negative
entK	Negative	Negative
entL	Negative	Negative
entM	Positive	Negative
entN (cons)	Positive	Negative

entN (other than RF122)	Positive	Negative
entO	Positive	Negative
egc (total)	Positive	Negative
entQ	Negative	Negative
entR	Negative	Negative
entU	Positive	Negative
entCM14 probe1	Negative	Negative
entCM14 probe2	Negative	Negative
lukF	Positive	Positive
lukS	Positive	Positive
lukS (ST22+ST45)	Ambiguous	Positive
hlgA	Positive	Positive
lukF-PV	Negative	Negative
lukS-PV	Negative	Negative
lukF-PV (P83)	Negative	Positive
lukM	Negative	Positive
lukD	Negative	Positive
lukE	Positive	Negative
lukX	Positive	Positive
lukY	Positive	Positive
lukY (ST30+ST45)	Negative	Negative
hl	Positive	Positive
hla	Positive	Positive
hlIII (cons)	Positive	Positive
hlIII (other than RF122)	Positive	Negative
hlb-probe 1	Ambiguous	Positive
hlb-probe 2	Positive	Positive
hlb-probe 3	Negative	Positive
un-truncated hlb	Positive	Negative
sak	Negative	Negative
chp	Negative	Negative
scn	Negative	Negative
etA	Negative	Negative
etB	Negative	Negative
etD	Negative	Negative
edinA	Negative	Negative
edinB	Negative	Negative
edinC	Negative	Negative
ACME (total)	Negative	Negative
arcA-SCC	Negative	Negative
arcB-SCC	Negative	Negative
arcC-SCC	Negative	Negative
arcD-SCC	Negative	Negative
aur (cons)	Positive	Positive
aur (cons)	Positive	Positive
aur (MRSA252)	Negative	Negative
splA	Positive	Positive
splB	Positive	Positive
splE	Negative	Negative
sspA	Positive	Positive
sspB	Positive	Positive
sspP (cons)	Positive	Positive
sspP (other than ST93)	Positive	Positive
setC	Positive	Positive
set6-var1_11	Negative	Negative
set6-var2_11	Positive	Positive
set6-var1_12	Negative	Negative
set6-var2_12	Positive	Positive
set6-var4_11	Negative	Negative
ssl01-RF122	Positive	Ambiguous

ssl01/set6 (COL)	Negative	Negative
ssl01/set6 (Mu50+N315)	Negative	Negative
ssl01/set6 (MW2+MSSA476)	Positive	Positive
ssl01/set6 (MRSA252)	Negative	Negative
ssl01/set6 (RF122)	Negative	Negative
ssl01/set6 (other alleles)	Negative	Negative
ssl02/set7	Positive	Positive
ssl02/set7 (MRSA252)	Negative	Ambiguous
ssl03/set8_probe 1	Ambiguous	Positive
ssl03/set8_probe 2	Ambiguous	Positive
ssl03/set8 (MRSA252, SAR0424)	Negative	Negative
ssl04/set9	Positive	Positive
ssl04/set9 (MRSA252, SAR0425)	Negative	Ambiguous
ssl05/set3_probe 1	Negative	Negative
ssl05/set3 (RF122, probe-611)	Ambiguous	Ambiguous
ssl05/set3_probe 2 (612)	Positive	Positive
ssl05/set3 (MRSA252)	Negative	Positive
ssl06/set21	Positive	Positive
ssl06 (NCTC8325+MW2)	Positive	Positive
ssl07/set1	Positive	Ambiguous
ssl07/set1 (MRSA252)	Ambiguous	Positive
ssl07/set1 (AF188836)	Ambiguous	Ambiguous
ssl08/set12_probe 1	Positive	Positive
ssl08/set12_probe 2	Positive	Positive
ssl09/set5_probe 1	Positive	Positive
ssl09/set5_probe 2	Positive	Positive
ssl09/set5 (MRSA252)	Negative	Negative
ssl10/set4	Positive	Positive
ssl10 (RF122)	Ambiguous	Ambiguous
ssl10/set4 (MRSA252)	Ambiguous	Ambiguous
ssl11/set2 (COL)	Negative	Negative
ssl11+set2(Mu50+N315)	Negative	Negative
ssl11+set2(MW2+MSSA476)	Negative	Negative
ssl11/set2 (MRSA252)	Negative	Negative
setB3	Positive	Positive
setB3 (MRSA252)	Negative	Ambiguous
setB2	Positive	Positive
setB2 (MRSA252)	Negative	Negative
setB1	Positive	Positive
cap 1	Negative	Negative
capH1	Negative	Negative
capJ1	Negative	Negative
capK1	Negative	Ambiguous
cap 5	Positive	Negative
capH5	Positive	Ambiguous
capJ5	Positive	Ambiguous
capK5	Positive	Negative
cap 8	Negative	Positive
capH8	Negative	Positive
capI8	Negative	Positive
capJ8	Negative	Positive
capK8	Negative	Positive
icaA	Positive	Positive
icaC	Positive	Positive
icaD	Positive	Positive
bap	Negative	Negative
bbp	Positive	Positive
bbp (cons)	Positive	Positive
bbp (COL+MW2)	Negative	Positive
bbp (MRSA252)	Negative	Negative

bbp (Mu50)	Negative	Ambiguous
bbp (RF122)	Negative	Ambiguous
bbp (ST45)	Negative	Ambiguous
clfA	Positive	Positive
clfA (cons)	Positive	Positive
clfA (COL+RF122)	Negative	Negative
clfA (MRSA252)	Negative	Negative
clfA (Mu50+MW2)	Positive	Positive
clfB	Positive	Positive
clfB (cons)	Positive	Positive
clfB (COL+Mu50)	Negative	Negative
clfB (MW2)	Negative	Negative
clfB (RF122)	Positive	Positive
cna	Negative	Negative
ebh (cons)	Positive	Positive
ebpS	Positive	Positive
ebpS_probe 612	Ambiguous	Negative
ebpS_probe 614	Positive	Positive
ebpS (01-1111)	Negative	Positive
ebpS (COL)	Positive	Ambiguous
eno	Positive	Positive
fib	Positive	Positive
fib (MRSA252)	Negative	Negative
fnbA	Positive	Positive
fnbA (cons)	Positive	Positive
fnbA (COL)	Negative	Negative
fnbA (MRSA252)	Positive	Negative
fnbA (Mu50+MW2)	Negative	Negative
fnbA (RF122)	Negative	Negative
fnbB	Negative	Positive
fnbB (COL)	Negative	Negative
fnbB (COL+Mu50+MW2)	Negative	Positive
fnbB (Mu50)	Negative	Negative
fnbB (MW2)	Negative	Ambiguous
fnbB (ST15)	Negative	Negative
fnbB (ST45-2)	Negative	Negative
map	Negative	Positive
map (COL)	Negative	Positive
map (MRSA252)	Negative	Negative
map (Mu50+MW2)	Negative	Positive
sasG	Positive	Negative
sasG (COL+Mu50)	Positive	Negative
sasG (MW2)	Negative	Ambiguous
sasG (OtherThan252+122	Positive	Negative
sdrC	Positive	Positive
sdrC (cons)	Positive	Positive
sdrC (B1)	Negative	Negative
sdrC (COL)	Negative	Positive
sdrC (Mu50)	Positive	Negative
sdrC (MW2+MRSA252+RF122)	Positive	Positive
sdrC (OtherThan252+RF122)	Positive	Negative
sdrD	Positive	Positive
sdrD (cons)	Positive	Positive
sdrD (COL+MW2)	Negative	Negative
sdrD (Mu50)	Negative	Positive
sdrD (other)	Positive	Negative
vwb	Positive	Positive
vwb (cons)	Positive	Positive
vwb (COL+MW2)	Negative	Negative
vwb (MRSA252)	Negative	Negative

vwb (Mu50)	Positive	Negative
vwb (RF122)	Negative	Negative
isaB	Positive	Positive
isaB (MRSA252)	Ambiguous	Ambiguous
mprF (COL+MW2)	Positive	Ambiguous
mprF (Mu50+252)	Ambiguous	Positive
isdA (cons)	Positive	Positive
isdA (MRSA252)	Negative	Ambiguous
isdA (Other Than MRSA252)	Positive	Positive
lmrP (OtherThanRF122)	Positive	Ambiguous
lmrP (OtherThanRF122)	Positive	Ambiguous
lmrP (RF122)	Negative	Positive
lmrP (RF122)	Negative	Positive
hsdS1-RF122	Negative	Negative
hsdS2-ST5+ST8	Negative	Negative
hsdS2-MW2+476	Positive	Ambiguous
hsdS2-RF122	Negative	Negative
hsdS2-MRSA252	Negative	Negative
hsdS3-AllOtherThanRF122+252	Negative	Positive
hsdS3-ST8+ST1+RF122	Negative	Negative
hsdS3-Mu50+N315	Negative	Negative
hsdS3-CC51+252	Negative	Positive
hsdS3-MRSA252	Negative	Negative
hsdSx-CC25	Negative	Positive
hsdSx-CC15	Negative	Positive
hsdSx-etd	Negative	Negative
Q2FXC0	Negative	Negative
Q2YUB3	Ambiguous	Positive
Q7A4X2	Positive	Positive
hysA1 (MRSA252)	Negative	Negative
hysA1 (MRSA252+RF122) and/or hysA2 (cons)	Positive	Positive
hysA1 (MRSA252+RF122) and/or hysA2 (COL+USA300)	Negative	Negative
hysA2 (All Other Than MRSA252)	Negative	Negative
hysA2 (COL+USA300+NCTC)	Negative	Negative
hysA2 (All Other Than COL+USA300+NCTC)	Positive	Positive
hysA2-AllOtherThan COL+USA300+NCTC	Positive	Positive
hysA2 (MRSA252)	Negative	Negative
