# Supplementary material for: Mastitis Pathogens with High Virulence in a Mouse Model Produce a Distinct Cytokine Profile In Vivo
Source: Front Immunol. 2016 Sep 22;7:368. doi: 10.3389/fimmu.2016.00368 (PMC5031784; doi:10.3389/fimmu.2016.00368)
Supplement: Supplementary file 2 [file Table_2.PDF]

Supplemental Table 2. Genotyping of *E. coli* clinical isolates; strain 676 and 127

	E. coli strain	
**Gene name**	**676**	**127**
K88ab_10	Negative	Negative
astA_consens_10	Negative	Negative
bfpA_10	Negative	Negative
cba_10	Negative	Negative
ccl_10	Negative	Negative
cdtB_40	Positive	Negative
cdtB_50	Positive	Negative
cdtB_60	Negative	Negative
celb_10	Negative	Negative
cfa_c_10	Negative	Negative
cma_20	Negative	Negative
cnf1_20	Positive	Negative
cofA_10	Negative	Negative
eae_consensus_10	Negative	Negative
eae_consensus_20	Negative	Negative
eae_consensus_30	Negative	Negative
eae_consensus_40	Negative	Negative
espB_O157_20	Negative	Negative
espB_O26_40	Negative	Negative
f17-A_40	Negative	Negative
f17-A_50	Positive	Negative
f17-A_60	Negative	Negative
f17-G_20	Positive	Negative
fanA_10	Negative	Negative
fasA_10	Negative	Negative
fedA_10	Negative	Negative
fedF_10	Negative	Negative
fim41a_10	Negative	Negative
gad_10	Positive	Positive
hlyA_20	Negative	Negative
hlyE_10	Negative	Negative
ipaD_10	Negative	Negative
ipaH9.8_20	Negative	Negative
ireA_20	Negative	Negative
iroN_10	Negative	Negative
iss_10	Negative	Negative
lngA_20	Negative	Negative
ltcA_20	Negative	Negative
mchB_10	Negative	Negative
mchC_20	Negative	Negative
mchF_10	Negative	Negative
mcmA_10	Positive	Negative
nfaE_10	Negative	Negative
perA_10	Negative	Negative
perA_20	Negative	Negative

pet_20	Negative	Negative
prfB_30	Positive	Negative
senB_20	Negative	Negative
sfaS_10	Negative	Negative
sta1_110	Negative	Negative
sta2_210	Negative	Negative
stb_10	Negative	Negative
stx1A_10	Negative	Negative
stx2A_10	Negative	Negative
virF_20	Negative	Negative
hp_cif_611	Negative	Negative
hp_eaaA_611	Negative	Negative
hp_eatA_611	Negative	Negative
hp_efa1_611	Negative	Negative
hp_epeA_611	Negative	Negative
hp_espA_Crod_611	Negative	Negative
hp_espA_O103H2_611	Negative	Negative
hp_espA_O119H6_611	Negative	Negative
hp_espA_O127H7_611	Negative	Negative
hp_espA_O157H11_611	Negative	Negative
hp_espA_O49H12_611	Negative	Negative
hp_espA_O55H7_611	Negative	Negative
hp_espA_O8_611	Negative	Negative
hp_espC_611	Negative	Negative
hp_espF_611	Negative	Negative
hp_espF_612	Negative	Negative
hp_espF_Crod_611	Negative	Negative
hp_espF_O103H2_611	Negative	Negative
hp_espF_O103H2_612	Negative	Negative
hp_espI_611	Negative	Negative
hp_espJ_611	Negative	Negative
hp_espJ_612	Negative	Negative
hp_espP_611	Negative	Negative
hp_etpD_611	Negative	Negative
hp_iha_611	Negative	Negative
hp_katP_611	Negative	Negative
hp_lpfA_611	Positive	Negative
hp_nleA_611	Negative	Negative
hp_nleA_612	Negative	Negative
hp_nleA_613	Negative	Negative
hp_nleA_614	Negative	Negative
hp_nleB_611	Negative	Negative
hp_nleB_O157H7_611	Negative	Negative
hp_nleB_Styp_611	Negative	Negative
hp_nleC_611	Negative	Negative
hp_pic_611	Negative	Negative
hp_rpeA_611	Negative	Negative
hp_saa_611	Negative	Negative
hp_sat_611	Negative	Negative
hp_sepA_611	Negative	Negative

hp_sigA_611	Negative	Negative
hp_stxA2_611	Negative	Negative
hp_stxA2_613	Negative	Negative
hp_stxA2_614	Negative	Negative
hp_stxA2_615	Negative	Negative
hp_stxA2_616	Negative	Negative
hp_stxA2_617	Negative	Negative
hp_stxA2_618	Negative	Negative
hp_stxB2_612	Negative	Negative
hp_stxB2_613	Negative	Negative
hp_stxB2_614	Negative	Negative
hp_stxB2_615	Negative	Negative
hp_subA_611	Negative	Negative
hp_tccP_611	Negative	Negative
hp_tccP_612	Negative	Negative
hp_tir_4051.6_611	Negative	Negative
hp_tir_MPEC_611	Negative	Negative
hp_tir_NTH19_611	Negative	Negative
hp_tir_O103H2_611	Negative	Negative
hp_tir_O111_611	Negative	Negative
hp_tir_O157H45_611	Negative	Negative
hp_tir_O157H7_611	Negative	Negative
hp_toxB_611	Negative	Negative
hp_toxB_612	Negative	Negative
hp_toxB_613	Negative	Negative
hp_tsh_611	Negative	Negative
hp_vat_611	Negative	Negative
prob_ihfA_611	Positive	Positive
prob_gapA_611	Positive	Positive
0,1M NaPP Standard pH 9	Negative	Negative
Biotin-Marke_2,5 才 M	Positive	Positive
