# Supplementary material for: Mastitis Pathogens with High Virulence in a Mouse Model Produce a Distinct Cytokine Profile In Vivo
Source: Front Immunol. 2016 Sep 22;7:368. doi: 10.3389/fimmu.2016.00368 (PMC5031784; doi:10.3389/fimmu.2016.00368)
Supplement: Supplementary file 3 [file Image_1.PDF]

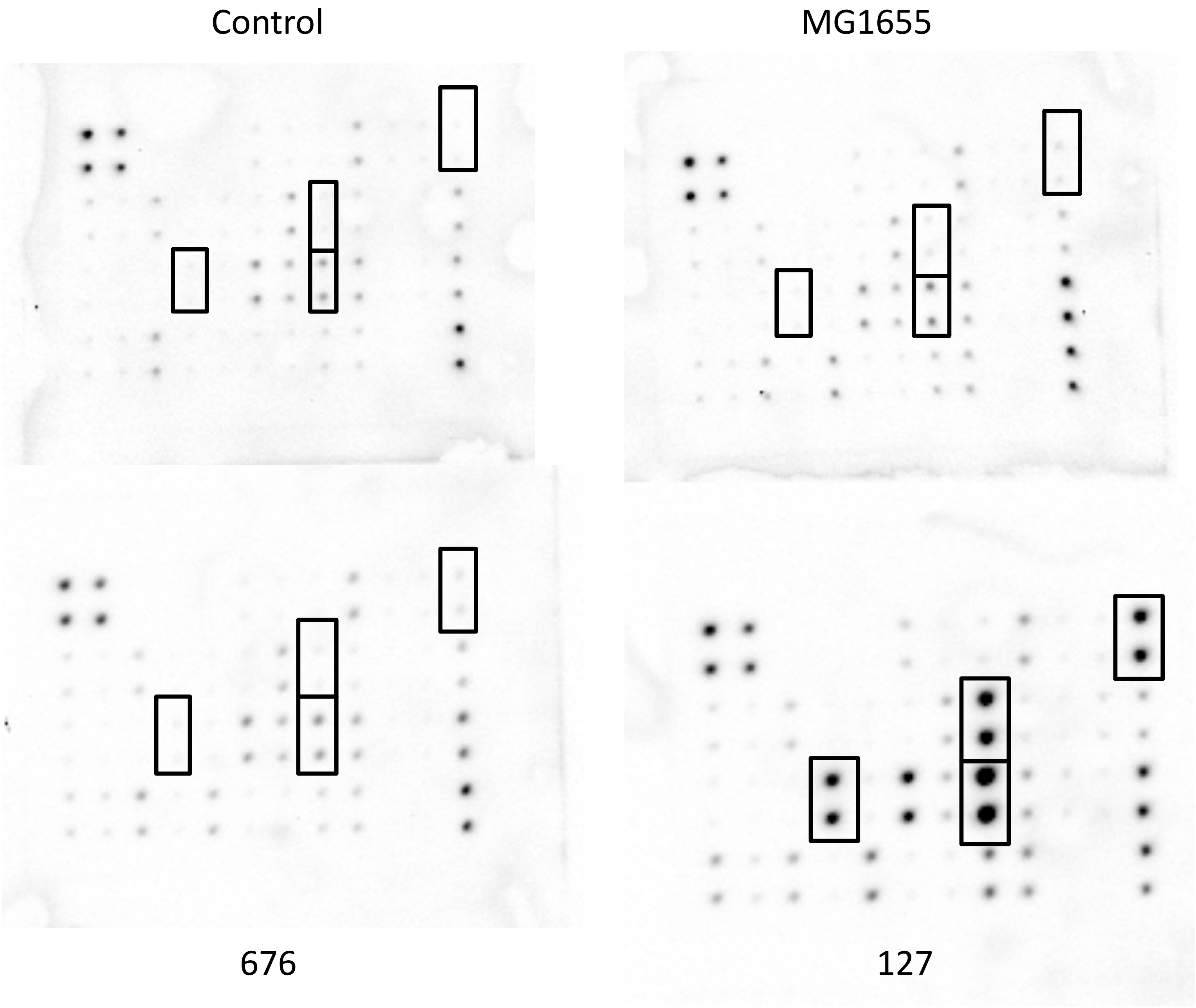

POS1	POS2	NEG	NEG	BLANK	BLC	CD30LG	CCL11	CCL24	FASLG	CX3CL1	G-CSF
POS1	POS2	NEG	NEG	BLANK	BLC	CD30LG	CCL11	CCL24	FASLG	CX3CL1	G-CSF
GM-CSF	IFN-γ	IL-1α	IL-1β	IL-2	IL-3	IL-4	IL-6	IL-9	IL-10	IL-12 p40p70	IL-12p70
GM-CSF	IFN-γ	IL-1α	IL-1β	IL-2	IL-3	IL-4	IL-6	IL-9	IL-10	IL-12 p40p70	IL-12p70
IL-13	IL-17A	I-TAC	CXCL1	Leptin	LIX	XCL1	CCL2	M-CSF	MIG	MIP-1α	MIP-1γ
IL-13	IL-17A	I-TAC	CXCL1	Leptin	LIX	XCL1	CCL2	M-CSF	MIG	MIP-1α	MIP-1γ
RANTES	SDF-1	TCA-3	TECK	TIMP1	TIMP2	TNF-α	sTNFR1	sTNFR2	BLANK	BLANK	POS
RANTES	SDF-1	TCA-3	TECK	TIMP1	TIMP2	TNF-α	sTNFR1	sTNFR2	BLANK	BLANK	POS

Suppl. Fig. 1
